# Supplementary material for: Programmed microalgae-gel promotes chronic wound healing in diabetes
Source: Nat Commun. 2024 Feb 3;15:1042. doi: 10.1038/s41467-024-45101-9 (PMC10838327; doi:10.1038/s41467-024-45101-9)
Supplement: Supplementary file 1 — Supplementary Information [file 41467_2024_45101_MOESM1_ESM.pdf]

Supplementary Information

Programmed Microalgae-Gel Promotes Chronic Wound Healing in Diabetes

Yong Kang[1,#], Lingling Xu[1,#], Jinrui Dong[1,#], Xue Yuan[1], Jiamin Ye[1], Yueyue Fan[1], Bing Liu[2,*], Julin Xie[3,*], Xiaoyuan Ji[1,4*]

[1] Academy of Medical Engineering and Translational Medicine, Medical College, Tianjin University, Tianjin 300072, China

[2] Department of Disease Control and Prevention， Rocket Force Characteristic Medical Center， Beijing 10088, China

[3] Department of Burns, The First Affiliated Hospital of Sun Yat-Sen University, Guangzhou 510080, China

[4] Medical College, Linyi University, Linyi 276000, China

*Corresponding authors:

neaucn@126.com (B. Liu), xiejulin@mail.sysu.edu.cn (J. Xie), jixiaoyuan@tju.edu.cn (X. Ji)

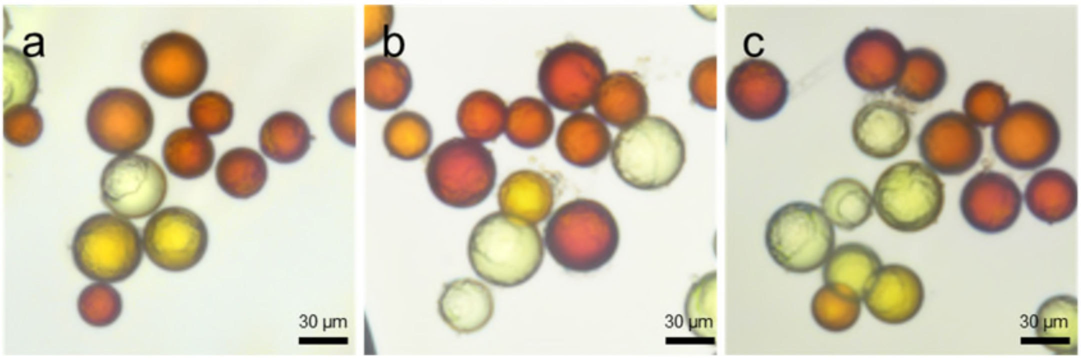

Supplementary Figure 1. The morphology of HEA protoplasts after incubated for (a) 0 h, (b) 12 h, and (c) 24 h. Each experiment was repeated three times independently with similar results.

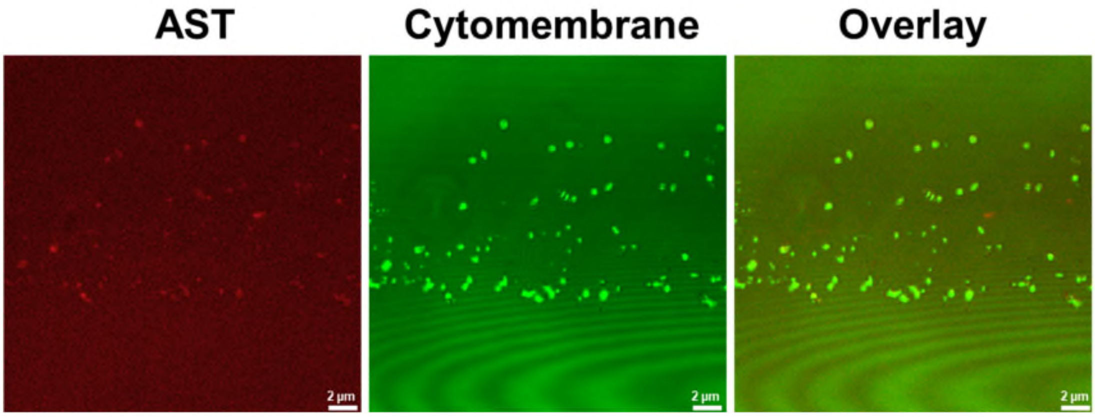

Supplementary Figure 2. Laser confocal microscope photographs for co-localization of AST and the vesicles. Each experiment was repeated three times independently with similar results.

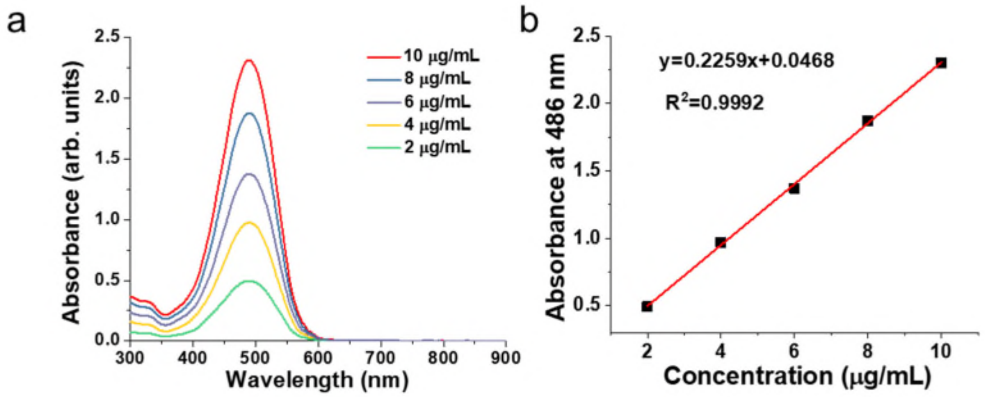

Supplementary Figure 3. (a) Absorbance of AST. (b) Normalized absorbance intensity of AST at 486 nm. Each experiment was repeated three times independently with similar results.

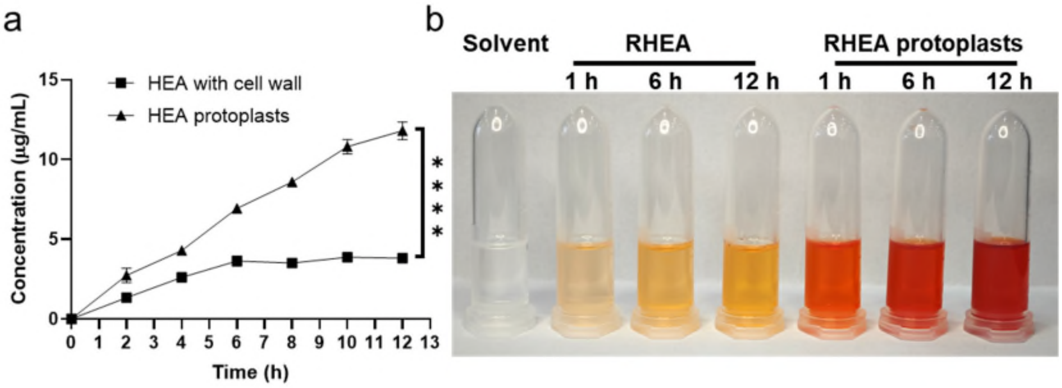

Supplementary Figure 4. (a) The amount of AST released over time for HEA protoplasts and HEA with cell walls. The data are presented as the mean ± s.d. (n = 3 biologically independent cells). (b) Photographs of AST solution released at different time points. Each experiment was repeated three times independently with similar results.

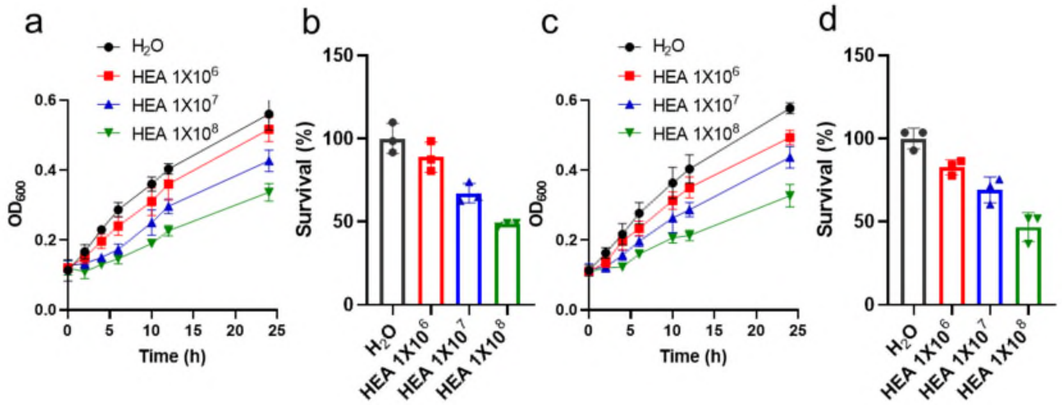

Supplementary Figure 5: (a, b) Quantitative measurement of E. coli cells treated with GHEA. Data are presented as mean ± s.d. (n = 3 biologically independent cells). (c, d) Quantitative measurement of S. aureus cells treated with GHEA. Data are presented as mean ± s.d. (n = 3 biologically independent cells).

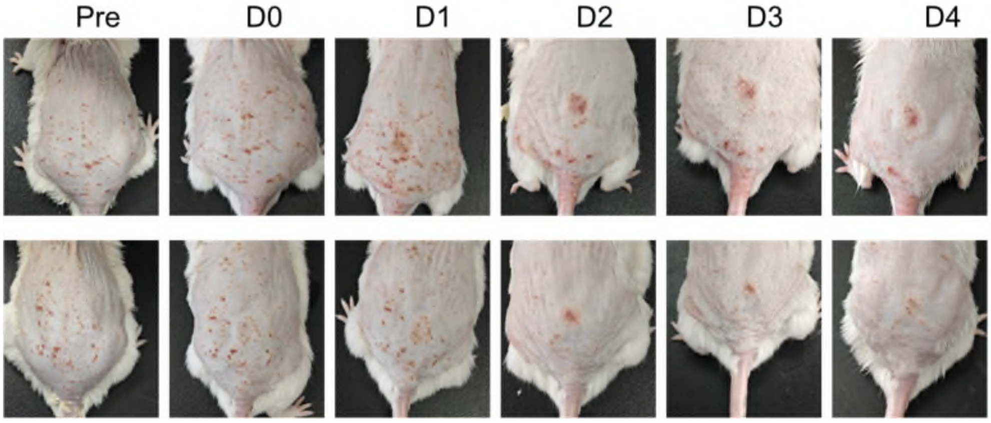

Supplementary Figure 6. Skin damage in mice at 55°C. Each experiment was repeated three times independently with similar results.

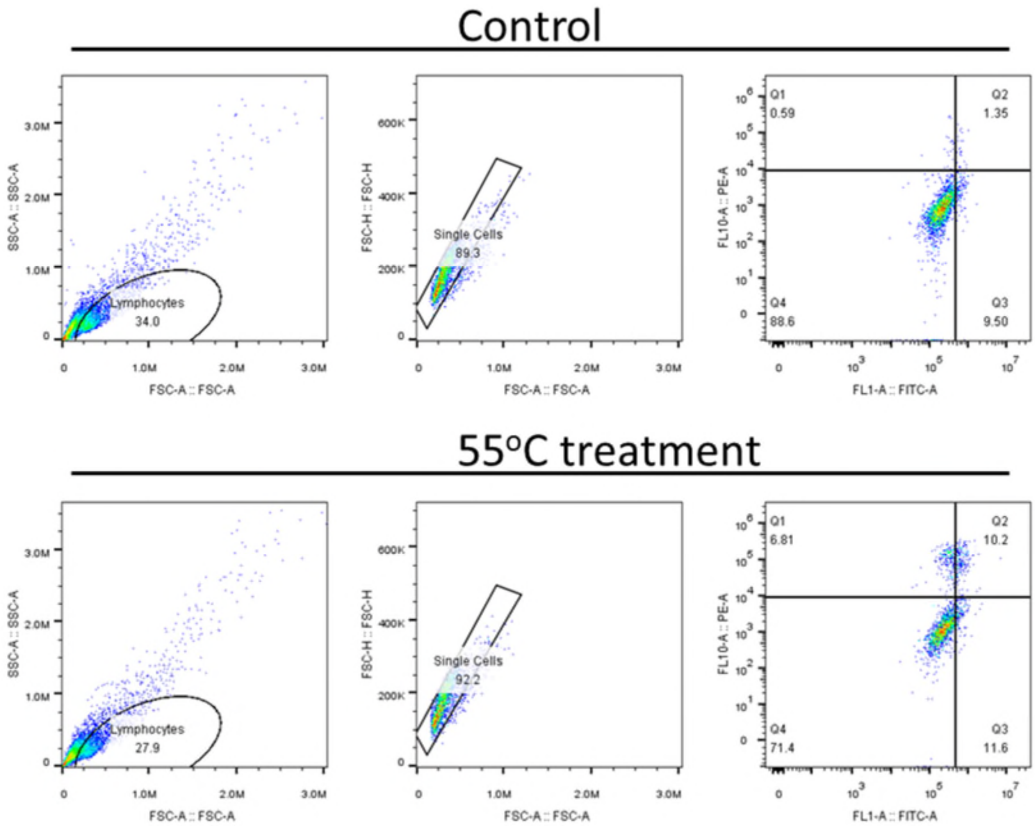

Supplementary Figure 7. Flow cytometry analysis of cells treated at 55°C.

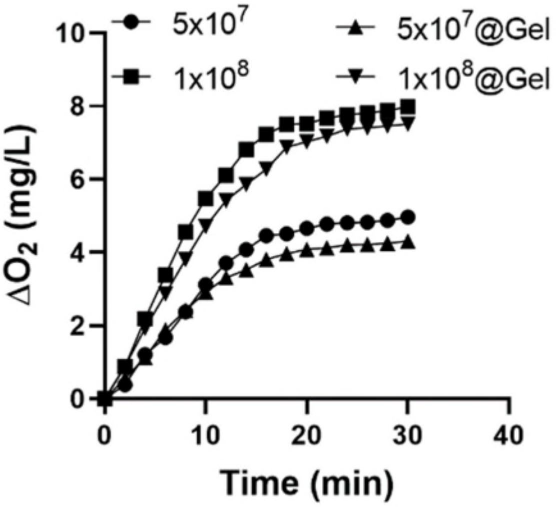

Supplementary Figure 8. The release of dissolved O_2 at various GHEA concentrations under 658 nm laser irradiation with an intensity of 0.5 W/cm^2. Each experiment was repeated three times independently with similar results.

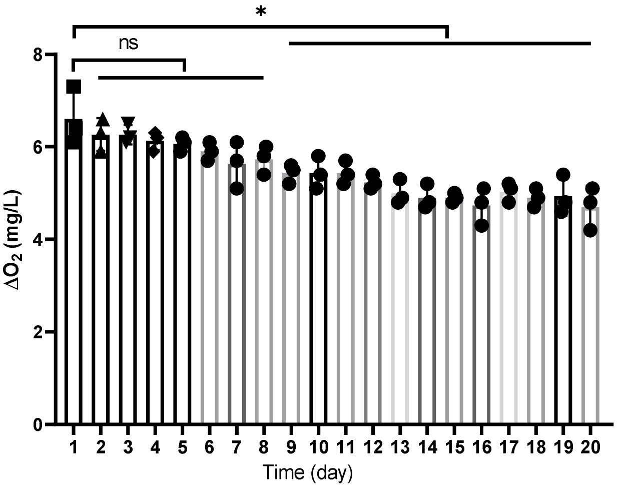

Supplementary Figure 9. The release of dissolved O_2 of GHEA during 20 days under laser irradiation. Data are presented as mean ± s.d. (n = 3 biologically independent cells).

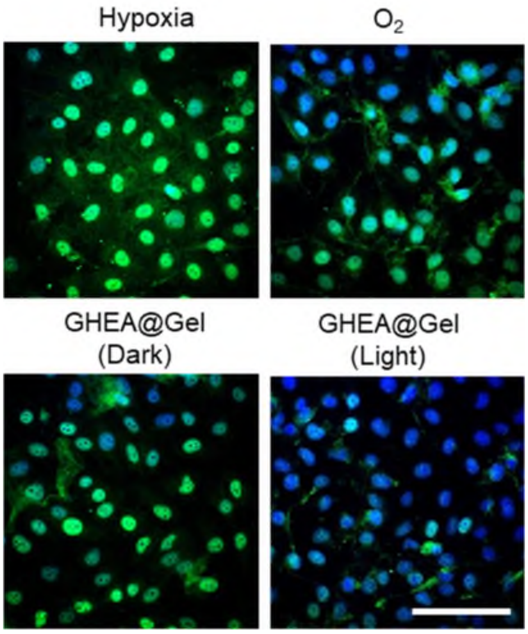

Supplementary Figure 10. The fluorescent images of GHEA@Gel reduced HIF-1α on high glucose–induced HUVECs. Scale bars, 100 μm. Three times each experiment was repeated independently with similar results.

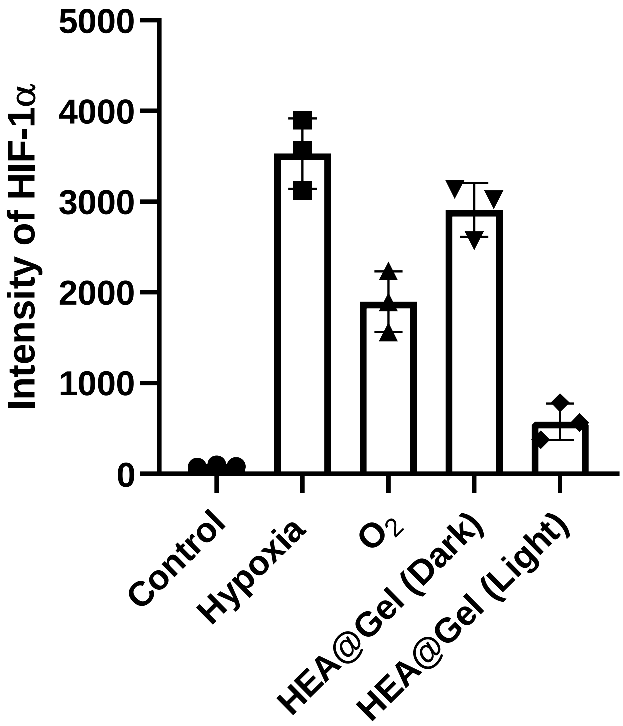

Supplementary Figure 11. The quantitative analysis of GHEA@Gel reduced HIF-1α on high glucose–induced HUVECs. Data are presented as mean ± s.d. (n = 3 independent cells).

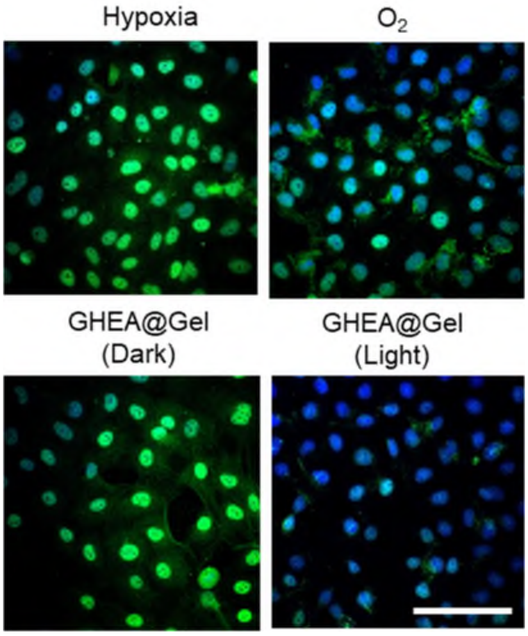

Supplementary Figure 12. The fluorescent images of GHEA@Gel reduced HIF-1α on high glucose–induced HaCaTs. Scale bars, 100 μm. Three times each experiment was repeated independently with similar results.

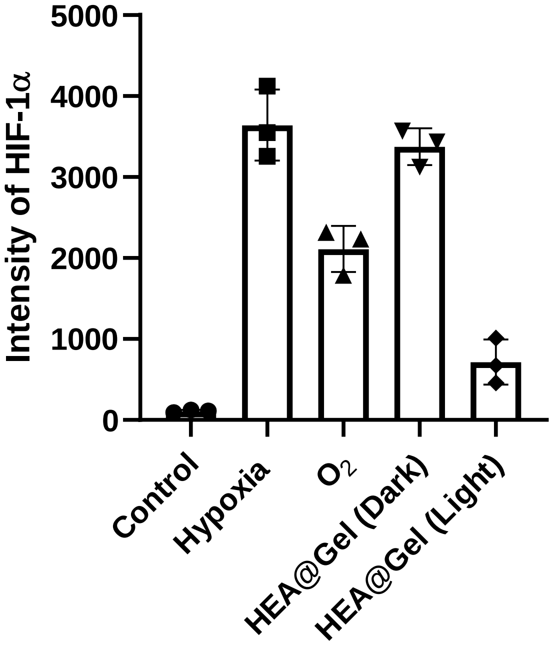

Supplementary Figure 13. The quantitative analysis of GHEA@Gel reduced HIF-1α on high glucose–induced HaCaTs. Data are presented as mean ± s.d. (n = 3 independent cells).

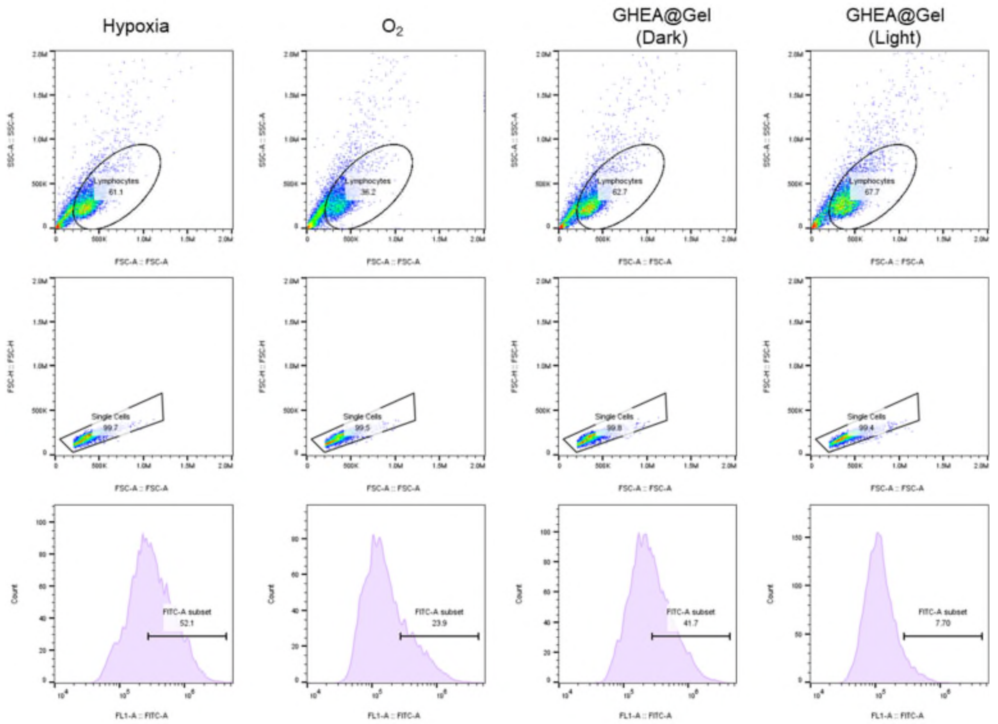

Supplementary Figure 14. The amount of intracellular HIF-1α expression in HSFs cells after treatment under different conditions and detection by FCM.

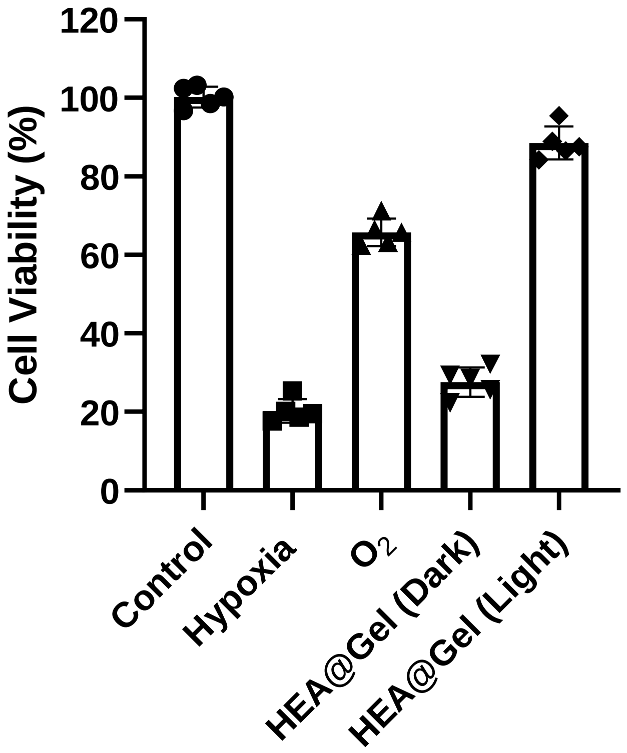

Supplementary Figure 15. HUVECs proliferation with 33 mM glucose and 6 hours of hypoxia in different groups. Data are presented as mean ± s.d. (n = 5 independent cells).

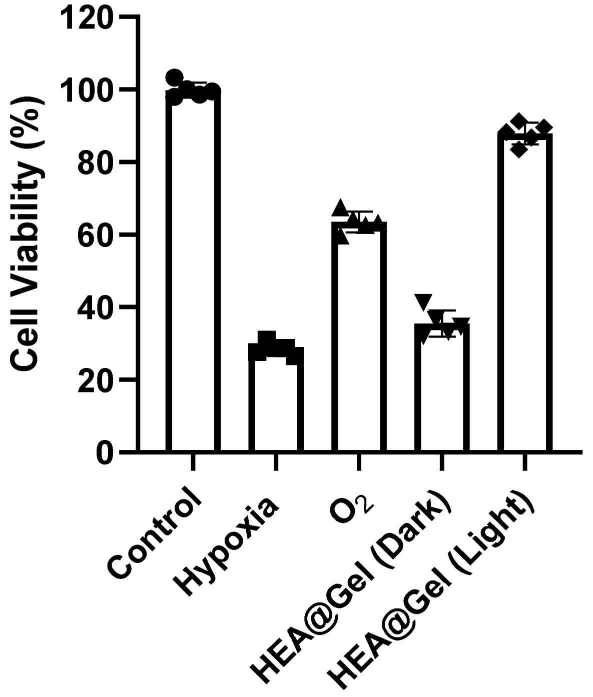

Supplementary Figure 16. HaCaTs proliferation with 33 mM glucose and 6 hours of hypoxia in different groups. Data are presented as mean ± s.d. (n = 5 independent cells).

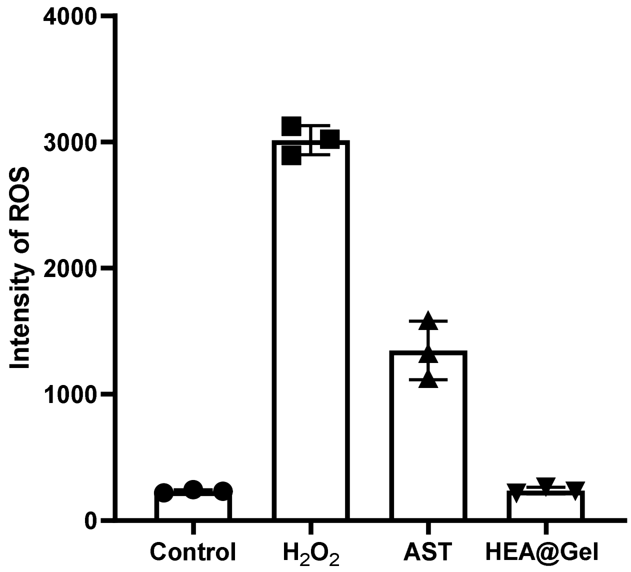

Supplementary Figure 17. ROS scavenging of RHEA@Gel in HUVECs. Data are presented as mean ± s.d. (n = 3 independent cells).

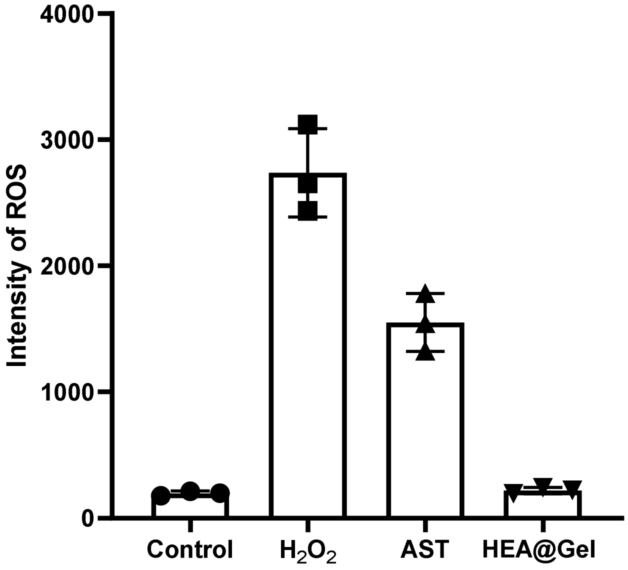

Supplementary Figure 18. ROS scavenging of RHEA@Gel in HaCaTs. Data are presented as mean ± s.d. (n = 3 independent cells).

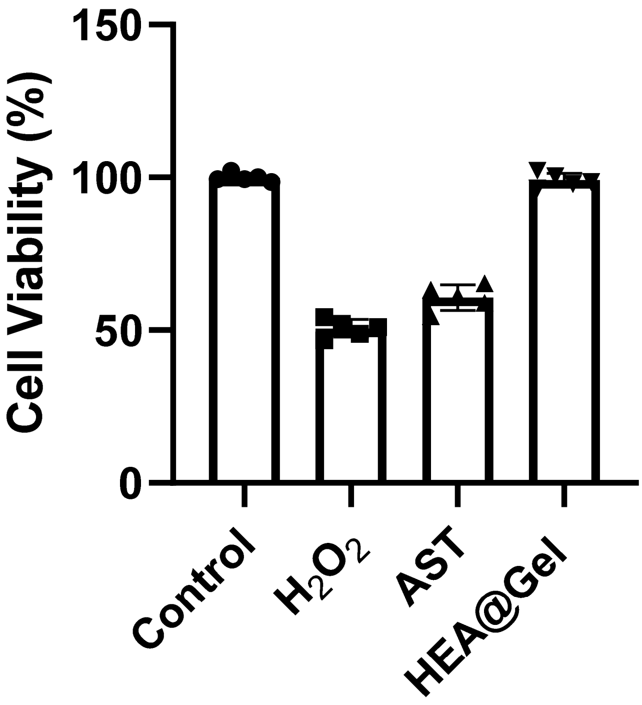

Supplementary Figure 19. HUVECs cell proliferation with 33 mM glucose and H_2O_2 in different groups. Data are presented as mean ± s.d. (n = 5 independent cells).

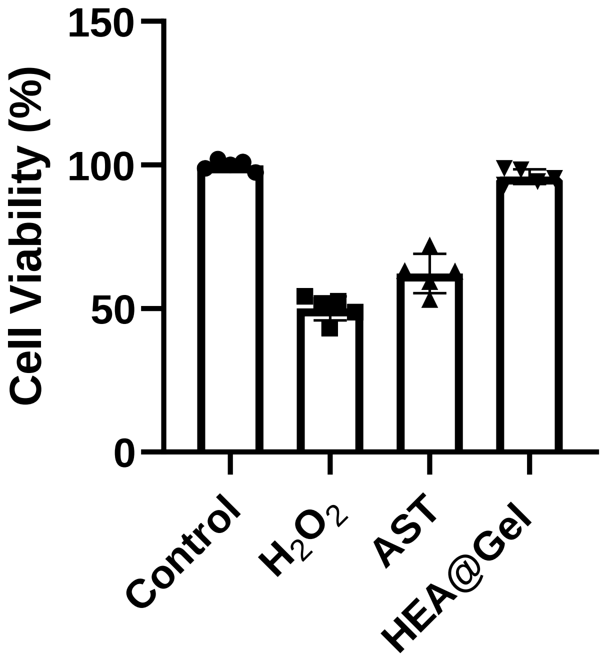

Supplementary Figure 20. HaCaTs cell proliferation with 33 mM glucose and H_2O_2 in different groups. Data are presented as mean ± s.d. (n = 5 independent cells).

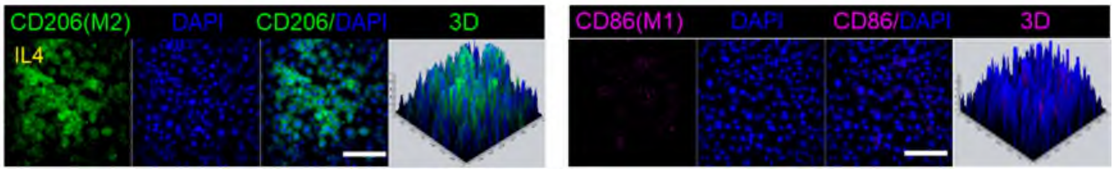

Supplementary Figure 21. Characteristic fluorescence images of Raw264. 7 cells with CD206 (green) and CD86 (pink) staining under IL4-stimulation. Three times each experiment was repeated independently with similar results. Scale bars, 100 μm.

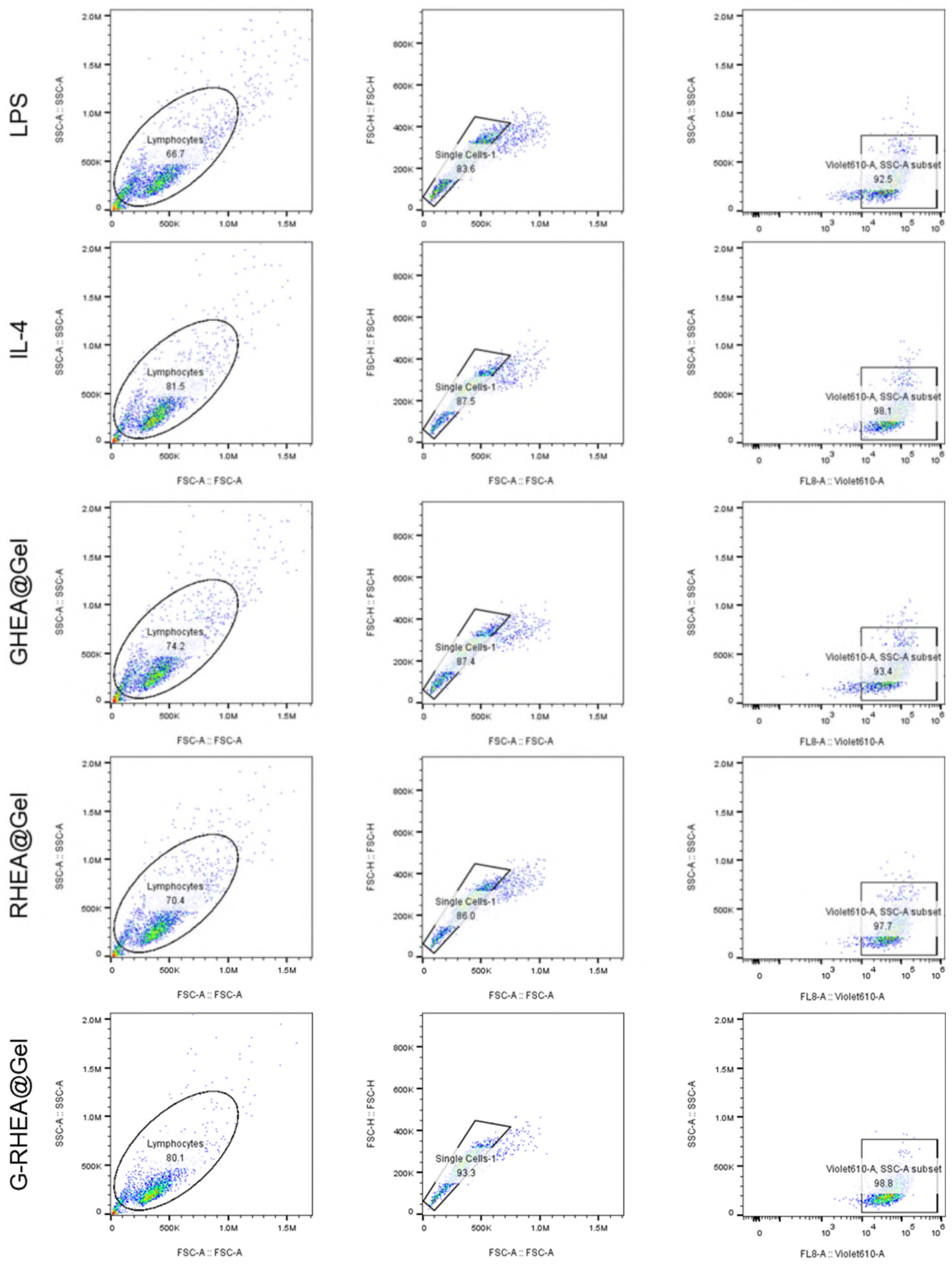

Supplementary Figure 22. Flow cytometry gating strategy for analysis of the polarization state of macrophages under different treatments in vitro in Figure 6d.

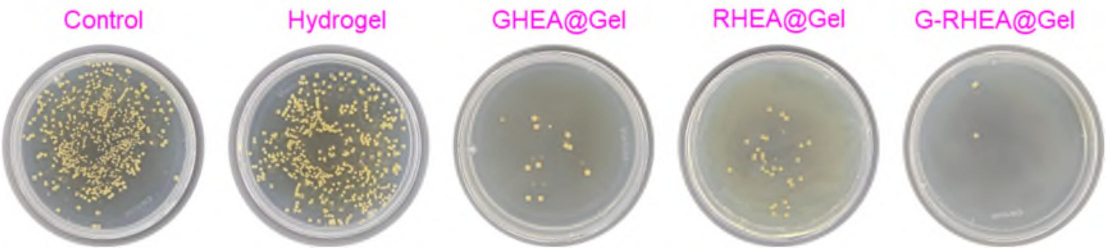

Supplementary Figure 23. The bacterial colonies of skin extract after different treatments. Three times each experiment was repeated independently with similar results.

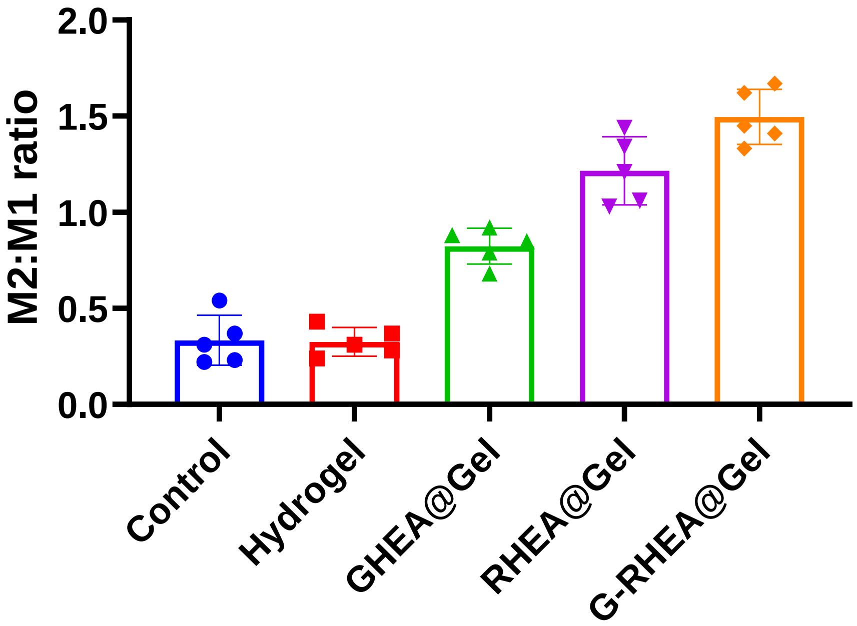

Supplementary Figure 24. The ratio of M2 and M1 under different treatments. Data are presented as mean ± s.d. (n = 5 independent mice).

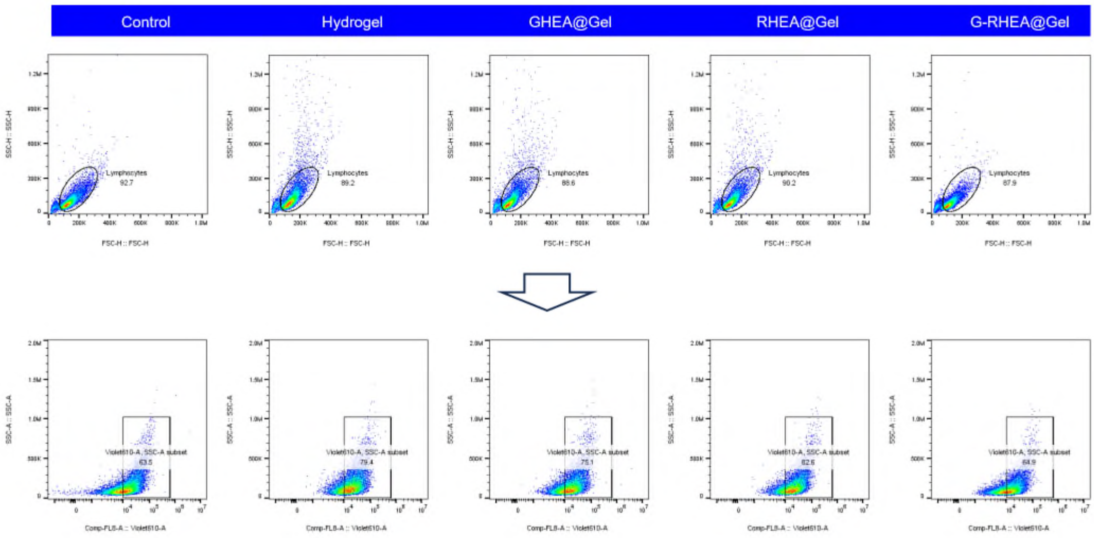

Supplementary Figure 25. Flow cytometry gating strategy for analysis of the polarization state of macrophages under different treatments in vivo in Figure 9b.

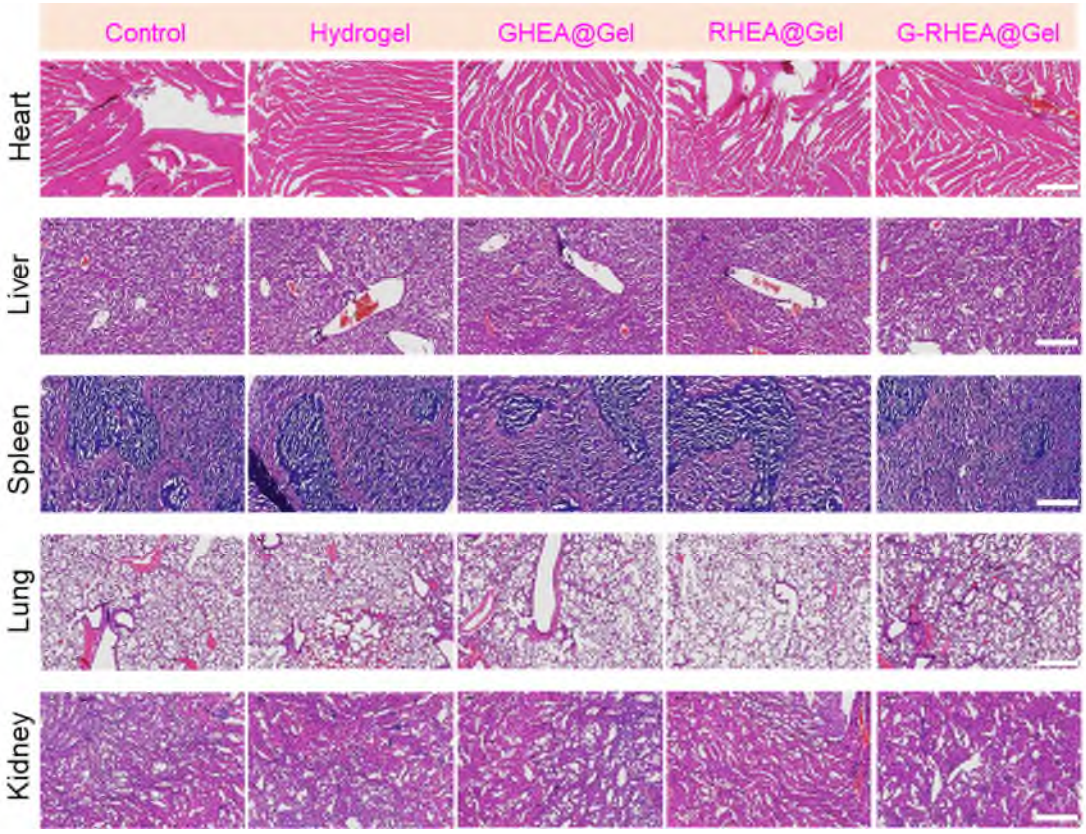

Supplementary Figure 26. H&E staining images of mice under different treatments. Three times each experiment was repeated independently with similar results. Scale bars, 500 μm.

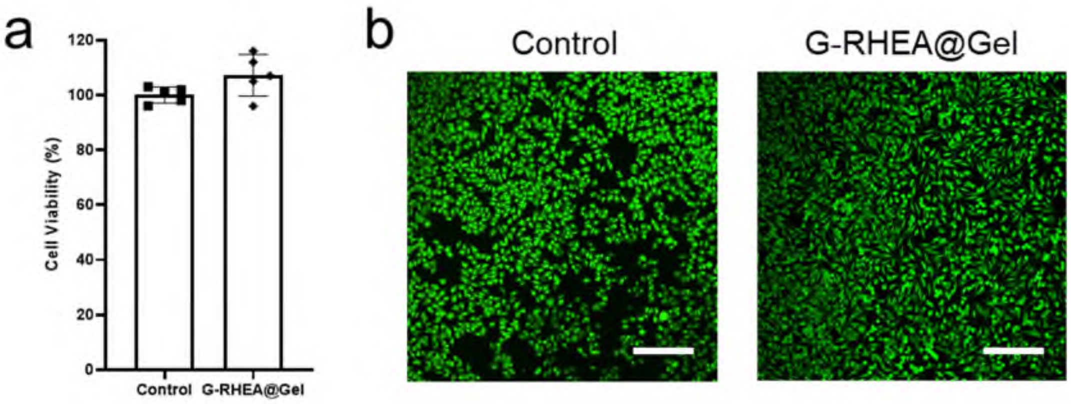

Supplementary Figure 27. (a) The biocompatibility of G-RHEA@Gel for HaCaTs was assessed by MTT assays. The data are presented as the mean ± s.d. (n = 5 biologically independent cells). (b) Live/dead staining images of HaCaTs treated with G-RHEA@Gel. Scale bars, 200 μm. Each experiment was repeated independently three times with similar results.

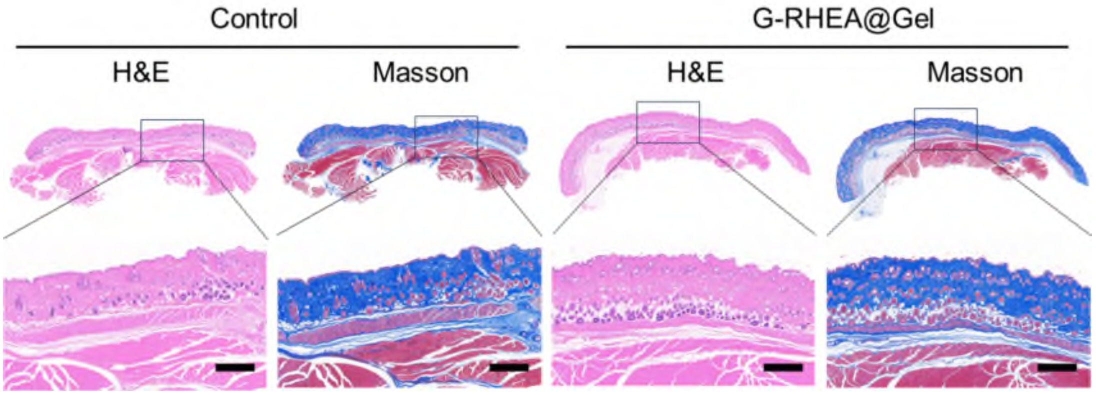

Supplementary Figure 28. H&E and Masson staining images of skin after different treatments. Scale bars, 500 μm. Each experiment was repeated independently three times with similar results.

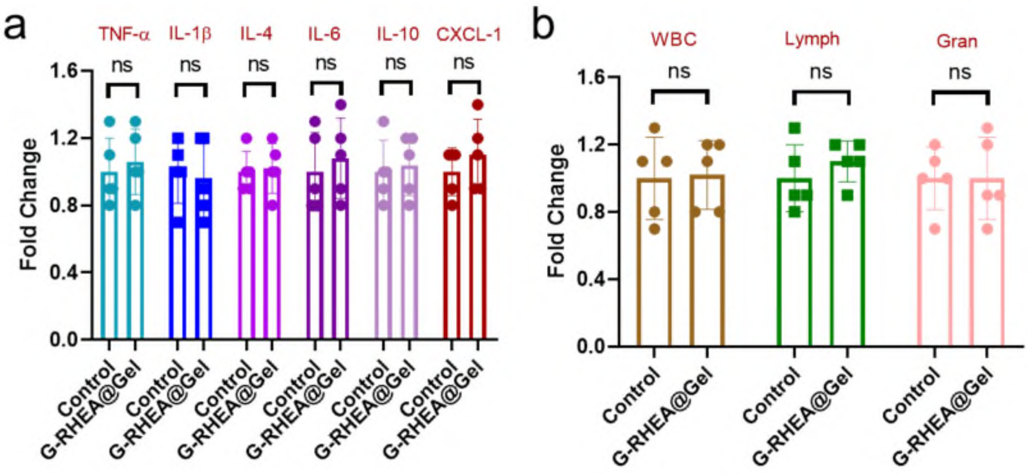

Supplementary Figure 29. (a) Analysis of inflammatory factors in the peripheral blood. Data are presented as the mean ± s.d. (n = 5 biologically independent mice). (b) Blood hematology analysis of the mic. Data are presented as the mean ± s.d. (n = 5 biologically independent mice). Statistical differences were analyzed by Student's two-sided t-test.
