# Supplementary material for: Programmed microalgae-gel promotes chronic wound healing in diabetes
Source: Nat Commun. 2024 Feb 3;15:1042. doi: 10.1038/s41467-024-45101-9 (PMC10838327; doi:10.1038/s41467-024-45101-9)
Supplement: Supplementary file 2 — Reporting Summary [file 41467_2024_45101_MOESM2_ESM.pdf]

nature portfolio

Corresponding author(s): Bing Liu, Julin Xie, Xiaoyuan Ji

Last updated by author(s): Jan 4, 2024

Reporting Summary

Nature Portfolio wishes to improve the reproducibility of the work that we publish. This form provides structure for consistency and transparency in reporting. For further information on Nature Portfolio policies, see our Editorial Policies and the Editorial Policy Checklist.

Statistics

For all statistical analyses, confirm that the following items are present in the figure legend, table legend, main text, or Methods section.

n/a	Confirmed	
☐	☒	The exact sample size (n) for each experimental group/condition, given as a discrete number and unit of measurement
☐	☒	A statement on whether measurements were taken from distinct samples or whether the same sample was measured repeatedly
☐	☒	The statistical test(s) used AND whether they are one- or two-sided<br>*Only common tests should be described solely by name; describe more complex techniques in the Methods section.*
☐	☒	A description of all covariates tested
☐	☒	A description of any assumptions or corrections, such as tests of normality and adjustment for multiple comparisons
☐	☒	A full description of the statistical parameters including central tendency (e.g. means) or other basic estimates (e.g. regression coefficient) AND variation (e.g. standard deviation) or associated estimates of uncertainty (e.g. confidence intervals)
☐	☒	For null hypothesis testing, the test statistic (e.g. F, t, r) with confidence intervals, effect sizes, degrees of freedom and P value noted<br>*Give P values as exact values whenever suitable.*
☒	☐	For Bayesian analysis, information on the choice of priors and Markov chain Monte Carlo settings
☐	☒	For hierarchical and complex designs, identification of the appropriate level for tests and full reporting of outcomes
☒	☐	Estimates of effect sizes (e.g. Cohen's d, Pearson's r), indicating how they were calculated

Our web collection on statistics for biologists contains articles on many of the points above.

Software and code

Policy information about availability of computer code

Data collection	BD FACSCalibur was used to collect flow cytometry data. TEM data was collected using JEM-2100UHR (JEOL, Japan). SEM data was collected using JSM-6700F (JEOL, Japan). Cryo-scanning electron microscopy data was collected using FEI Quanta 450 (USA). Infrared thermometer (TiS40 Infrared Camera FLUKE-TiS40 9Hz, FLUKE, USA) was used to collect photothermal pictures. Microscope pictures and fluorescent cell imaging were performed on KEYENCE BA-X700 (all-in-one Fluorescence Microscope). MTT data was collected using Bio-Rad Mode 680 microplate reader.
Data analysis	Anal of flow cytometry data was performed with Flowjo v7.6 software. Graph Pad Prism (8.0) and Origin 9.0 were used for data statistics and statistical significance calculation. Microsoft Excel 2016 was used for biodistribution and tumor size analysis. FL images were analyzed using Image J 1.8.0. Zetasizer Nano software v3.30 for analyses of particle size. TEM data was analyzed using Gatan-DigitalMicrograph-3.9.

For manuscripts utilizing custom algorithms or software that are central to the research but not yet described in published literature, software must be made available to editors and reviewers. We strongly encourage code deposition in a community repository (e.g. GitHub). See the Nature Portfolio guidelines for submitting code & software for further information.

Data

Policy information about availability of data

All manuscripts must include a data availability statement. This statement should provide the following information, where applicable:

- Accession codes, unique identifiers, or web links for publicly available datasets
- A description of any restrictions on data availability
- For clinical datasets or third party data, please ensure that the statement adheres to our policy

The authors declare that all data supporting the findings of this study are available within the article and the Supplementary Information. The full image dataset is available from the corresponding author upon request. Source data are provided with this paper.

Research involving human participants, their data, or biological material

Policy information about studies with human participants or human data. See also policy information about sex, gender (identity/presentation), and sexual orientation and race, ethnicity and racism.

Reporting on sex and gender	*Use the terms sex (biological attribute) and gender (shaped by social and cultural circumstances) carefully in order to avoid confusing both terms. Indicate if findings apply to only one sex or gender; describe whether sex and gender were considered in study design; whether sex and/or gender was determined based on self-reporting or assigned and methods used. Provide in the source data disaggregated sex and gender data, where this information has been collected, and if consent has been obtained for sharing of individual-level data; provide overall numbers in this Reporting Summary. Please state if this information has not been collected. Report sex- and gender-based analyses where performed, justify reasons for lack of sex- and gender-based analysis.*
Reporting on race, ethnicity, or other socially relevant groupings	*Please specify the socially constructed or socially relevant categorization variable(s) used in your manuscript and explain why they were used. Please note that such variables should not be used as proxies for other socially constructed/relevant variables (for example, race or ethnicity should not be used as a proxy for socioeconomic status). Provide clear definitions of the relevant terms used, how they were provided (by the participants/respondents, the researchers, or third parties), and the method(s) used to classify people into the different categories (e.g. self-report, census or administrative data, social media data, etc.) Please provide details about how you controlled for confounding variables in your analyses.*
Population characteristics	*Describe the covariate-relevant population characteristics of the human research participants (e.g. age, genotypic information, past and current diagnosis and treatment categories). If you filled out the behavioural & social sciences study design questions and have nothing to add here, write "See above."*
Recruitment	*Describe how participants were recruited. Outline any potential self-selection bias or other biases that may be present and how these are likely to impact results.*
Ethics oversight	*Identify the organization(s) that approved the study protocol.*

Note that full information on the approval of the study protocol must also be provided in the manuscript.

Field-specific reporting

Please select the one below that is the best fit for your research. If you are not sure, read the appropriate sections before making your selection.

☒ Life sciences ☐ Behavioural & social sciences ☐ Ecological, evolutionary & environmental sciences

For a reference copy of the document with all sections, see nature.com/documents/nr-reporting-summary-flat.pdf

Life sciences study design

All studies must disclose on these points even when the disclosure is negative.

Sample size	Sample size was chosen to ensure reproducibility of the experiments in accordance with the replacement, reduction and refinement principles of animal ethics regulation. Sample size are provided in the figure legends for each experiment and reasonable sample sizes were chosen to ensure they are sufficient for statistical comparison between different groups.
Data exclusions	No data were excluded from the analysis.
Replication	Experiments were repeated at least three independent experiments with similar results. All experiments were reproduced to reliably support conclusions stated in the manuscript.
Randomization	Animals were randomly distributed into the control and experimental groups. Each specific treatment was administrated to animals according to established schedules and regimens.
Blinding	Investigators were blinded when grouping mice of infected diabetic wound, performing biodistribution and imaging study. Investigators for

TEM and SEM characterization are blinded to the samples. During the experiments designed to evaluate therapeutic efficacy, animals model of infected diabetic wound was created and randomly divided into control and treatment groups. The investigators were blinded during these pre-clinical proof-of-concept studies based on combinatorial schemes.

Reporting for specific materials, systems and methods

We require information from authors about some types of materials, experimental systems and methods used in many studies. Here, indicate whether each material, system or method listed is relevant to your study. If you are not sure if a list item applies to your research, read the appropriate section before selecting a response.

Materials & experimental systems

n/a	Involved in the study
☐	☒ Antibodies
☐	☒ Eukaryotic cell lines
☒	☐ Palaeontology and archaeology
☐	☒ Animals and other organisms
☒	☐ Clinical data
☒	☐ Dual use research of concern
☒	☐ Plants

Methods

n/a	Involved in the study
☒	☐ ChIP-seq
☐	☒ Flow cytometry
☒	☐ MRI-based neuroimaging

Antibodies

Antibodies used

Mouse momoclonal Anti-CD86 antibody, Abcam (Product # 130-122-1), Dilution 1:200;
Mouse momoclonal Anti-CD206 antibody, Abcam (Product # 141706), Dilution 1:200;
Mouse momoclonal Anti-VEGF antibody, Santa Cruzse(Product # 57496), Dilution 1:200;
Mouse momoclonal Anti-EGF antibody, Abcam (Product # EPR19173), Dilution 1:500;
Mouse momoclonal Anti-HIF-1 alpha antibody, Abcam (Product # EP1215Y), Use a concentration of 0.5 µg/mL;
Mouse momoclonal Anti-CD31 antibody, Abcam (Product # 222783), Dilution 1:1000;
Mouse momoclonal Anti-alpha smooth muscle Actin (SMA) antibody, Abcam (Product # ab7817), Dilution 1:500;
Mouse momoclonal Anti-VIM antibody, Absin (Product # abs136555), Dilution 1:1000;
Mouse momoclonal Anti-COL antibody, Santa Cruzsc (Product # sc-59772), Dilution 1:500;
Rabbit polyclonal Anti-Staphylococcus aureus antibody, Absin (Product # ab20920), Dilution 1:1000;
Goat Anti-Rabbit IgG (H+L) Highly Cross-Adsorbed Secondary Antibody, ThermoFisher (Catalog # A-11034), Dilution 1:1000;
Goat Anti-Mouse IgG (H+L) Highly Cross-Adsorbed Secondary Antibody, ThermoFisher (Catalog # A-21236), Dilution 1:1000.

Validation

Abcam claims that Mouse momoclonal Anti-CD86 antibody is suitable for mice and IHC;
Abcam claims that Mouse momoclonal Anti-CD206 antibody is suitable for mice and IHC;
Santa Cruzsc claims that Mouse momoclonal Anti-VEGF antibody is suitable for mice and IHC;
Abcam claims that Mouse momoclonal Anti-EGF antibody is suitable for mice and IHC;
Abcam claims that Mouse momoclonal Anti-HIF-1 alpha antibody is suitable for mice and IHC;
Abcam claims that Anti-alpha smooth muscle Actin (SMA) antibody is suitable for mice and IHC;
Abcam claims that Mouse momoclonal Anti-CD31 antibody is suitable for mice and IHC;
Absin claims that Mouse momoclonal Anti-VIM antibody is suitable for mice and IHC;
Santa Cruzsc claims that Mouse momoclonal Anti-COL antibody is suitable for mice and IHC;
Absin claims that Rabbit polyclonal Anti-Staphylococcus aureus antibody is suitable for mice and IHC;
ThermoFisher claims that Goat Anti-Rabbit IgG (H+L) Highly Cross-Adsorbed Secondary Antibody is suitable for mice and IHC;
ThermoFisher claims that Goat Anti-Mouse IgG (H+L) Highly Cross-Adsorbed Secondary Antibody is suitable for mice and IHC;

Eukaryotic cell lines

Policy information about cell lines and Sex and Gender in Research

Cell line source(s)

Human skin fibroblasts (HSF, catalog number:PCS-201-012), human embryonic kidney cells (RAW264.7, catalog number: TIB-71), human umbilical vein endothelial cells(HUVEC, catalog number:PCS-100-013) and human immortalized keratinocytes (HaCaT, catalog number:PCS-200-011) were obtained from the American Type Culture Collections (ATCC).

Authentication

ATCC used morphology, karyotyping, and PCR based approaches to confirm the identity of human cell lines and to rule out both intra- and interspecies contamination. Also, the cell line were frequently checked by their morphological features.

Mycoplasma contamination

All cells were negative for mycoplasma.

Commonly misidentified lines
(See ICLAC register)

No commonly misidentified cell line were used.

Animals and other research organisms

Policy information about studies involving animals; ARRIVE guidelines recommended for reporting animal research, and Sex and Gender in Research

Laboratory animals	6 to 8-week-old female Balb/c mice (purchased from Tianjin Medical Laboratory Animal Center, Tianjin, China) were used in this study. The cages were placed in conventional rooms with controlled photoperiod (07:00-19:00 h white light, ± 200 lx at 1 m above the floor; 19:00-07:00 h red light, ± 5 lx at 1 m), temperature (20-22 °C), relative humidity (50-60%) and ventilation (15 air changes h-1).
Wild animals	No wild animals were used in this study.
Reporting on sex	The findings apply to all genders
Field-collected samples	This study did not involve samples collected from the fields.
Ethics oversight	All animal experiments were conducted according to the Guidelines for the Care and Use of Laboratory Animals of Tianjin University and were approved by the Animal Ethics Committee of the Tianjin University Laboratory Animal Center (Tianjin, China). The maximal tumour size permitted by Animal Ethics Committee of the Tianjin University Laboratory Animal Center is 2000 mm3.

Note that full information on the approval of the study protocol must also be provided in the manuscript.

Flow Cytometry

Plots

Confirm that:

- [x] The axis labels state the marker and fluorochrome used (e.g. CD4-FITC).
- [x] The axis scales are clearly visible. Include numbers along axes only for bottom left plot of group (a 'group' is an analysis of identical markers).
- [x] All plots are contour plots with outliers or pseudocolor plots.
- [x] A numerical value for number of cells or percentage (with statistics) is provided.

Methodology

Sample preparation	Single cell suspensions from cultured cells were used for flow cytometry tests.
Instrument	BD FACSCalibur
Software	Flowjo v7.6
Cell population abundance	*Describe the abundance of the relevant cell populations within post-sort fractions, providing details on the purity of the samples and how it was determined.*
Gating strategy	Generally, cells were first gated on FSC/SCC. Singlet cells were usually gated using FSC-H and FSC-A. Debris were removed by thresholding.

- [x] Tick this box to confirm that a figure exemplifying the gating strategy is provided in the Supplementary Information.
